# Generalist Life Cycle Aids Persistence of *Alexandrium ostenfeldii* (Dinophyceae) in Seasonal Coastal Habitats of the Baltic Sea

**DOI:** 10.1111/jpy.12919

**Published:** 2019-10-06

**Authors:** Jacqueline Jerney, Salla Annika Ahonen, Päivi Hakanen, Sanna Suikkanen, Anke Kremp

**Affiliations:** ^1^ Marine Research Center Finnish Environment Institute Helsinki 00790 Finland; ^2^ Tvärminne Zoological Station University of Helsinki Hanko 10900 Finland; ^3^ Leibniz‐Institut für Ostseeforschung Warnemünde Rostock 18119 Germany

**Keywords:** Cyst, dinoflagellate, dormancy, encystment, germination, microalgae, quiescence, resting cyst, sexual reproduction

## Abstract

In seasonal environments, strong gradients of environmental parameters can shape life cycles of phytoplankton. Depending on the rate of environmental fluctuation, specialist or generalist strategies may be favored, potentially affecting life cycle transitions. The present study examined life cycle transitions of the toxin producing Baltic dinoflagellate *Alexandrium ostenfeldii* and their regulation by environmental factors (temperature and nutrients). This investigation aimed to determine whether genetic recombination of different strains is required for resting cyst formation and whether newly formed cysts are dormant. Field data (temperature and salinity) and sediment surface samples were collected from a site with recurrent blooms and germination and encystment experiments were conducted under controlled laboratory conditions. Results indicate a lack of seasonal germination pattern, set by an endogenous rhythm, as commonly found with other dinoflagellates from the Baltic Sea. Germination of quiescent cysts was triggered by temperatures exceeding 10°C and combined nutrient limitation of nitrogen and phosphorus or a drop in temperature from 16 to 10°C triggered encystment most efficiently. Genetic recombination was not mandatory for the formation of resting cysts, but supported higher numbers of resistant cysts and enhanced germination capacity after a resting period. Findings from this study confirm that *A. ostenfeldii* follows a generalist germination and cyst formation strategy, driven by strong seasonality, which may support its persistence and possibly expansion in marginal environments in the future, if higher temperatures facilitate a longer growth season.

**Abbreviations**
a: *Alexandrium ostenfeldii* strain AOB202
ab: combination of two *Alexandrium ostenfeldii* strains: AOB202 and AOB348
ac: combination of two *Alexandrium ostenfeldii* strains: AOB202 and AOB504
b: *Alexandrium ostenfeldii* strain AOB348
bc: combination of two *Alexandrium ostenfeldii* strains: AOB348 and AOB504
c: *Alexandrium ostenfeldii* strain AOB504
C: control
mix: combination of five *Alexandrium ostenfeldii* strains: AOB202, AOB348, AOB504, AOB413, and AOB329
N: nitrogen limitation
NP: nitrogen and phosphorus limitation
P: phosphorus limitation
T: drop of temperature from 16 to 10°C

## Introduction

Life in the Baltic Sea must cope with strong seasonal fluctuations of environmental factors, such as temperature, light, and nutrients. When environmental conditions become unsuitable for growth, organisms must respond accordingly to endure. As part of many phytoplankton species’ life cycle, resting cysts serve as a crucial component for survival (Ellegaard and Ribeiro 2018). Particularly in environments with a strong seasonality, where vegetative growth may be restricted to a certain time of the year, the recurrence and timing of species in the water column can largely depend on the formation and germination of resting cysts (e.g., Kremp et al. 2009 and references therein). Since benthic sediments in coastal areas can be rich in resting cysts, they have important implications for plankton dynamics (e.g., Lundholm et al. 2011). Large deposits of long‐term resting cysts in the sediment—so‐called “seed banks”—were, for example, proposed to have an anchoring effect on aquatic microalgae populations, leading to increased genetic diversity and population differentiation (Sundqvist et al. 2018). Seed banks and benthic–pelagic coupling play a key role in harmful algal bloom formation, as they can lead to both locally restricted and large‐scale regional blooms (Anderson et al. 2012).

Diverse phylogenetic phytoplankton groups form various types of resting stages with different purposes in the life cycle. Some resting stages, such as the hypnozygotes of dinoflagellates may be linked to sexual reproduction, whereas other cysts are asexual and may be formed as a response to parasite infection, grazing pressure, or environmental cues, which signal the end of the growth period (Anderson 1998, Anderson et al. 2003, Toth et al. 2004, Ellegaard and Ribeiro 2018). Although the formation of phytoplankton resting cysts plays a crucial role for their ecology and evolution (Ellegaard and Ribeiro 2018), requirements and triggers of their formation and germination remain unknown for many taxa. This information is available for ecologically important and toxic dinoflagellate species, where detailed studies on life cycle transitions have significantly contributed to a better understanding of bloom dynamics (Anderson 1998, Bravo et al. 2010, Ní Rathaille and Raine 2011). These studies indicate that habitat‐specific environmental conditions require specific germination and encystment patterns, thereby shaping bloom strategies (Anderson 1998). In strongly seasonal habitats, environmental variability is high and suitable growth conditions can be restricted to a short time period. Germination outside of this time period can be inhibited by dormancy, a well‐documented trait that helps microorganisms cope with environmental variability (Lennon and Jones 2011). “Dormancy” is defined as suspension of growth by endogenous regulation, requiring a period of physiological ripening (maturation or dormancy period), before cysts can germinate (e.g., Anderson 1998). Once released from dormancy cysts may enter a state of “quiescence,” which is defined as suspension of growth due to external conditions (e.g., environmental factors; Anderson 1998). Internal (e.g., endogenous clock, or other) and/or external (e.g., temperature) factors regulate dormancy transitions, and when cysts are quiescent, certain temperature ranges can stimulate germination (Anderson and Rengefors 2006, Fischer et al. 2018). Temperature acts as a reliable signal by changing in predictable ways during the seasonal cycle, thereby setting the window for growth, depending on growth requirements of the respective species (Ellegaard and Ribeiro 2018). While dormancy and temperature primarily drive germination of seasonal dinoflagellates, cyst formation often depends on nutrient conditions, particularly when linked to sexual reproduction (Figueroa et al. 2008). The blooms of cold water dinoflagellates commonly occurring in the northern Baltic Sea during spring are regulated by dormancy, temperature, and nutrient‐dependent life cycle transitions (Kremp et al. 2009).

Since the early 2000s, the toxin producing dinoflagellate *Alexandrium ostenfeldii* (Paulsen) Balech and Tangen (1985) has started to form dense, recurrent blooms during summer in shallow coastal waters of the Baltic Sea (Kremp et al. 2009). Like most other *Alexandrium* species, *A. ostenfeldii* forms resting cysts (Mackenzie et al. 1996, Anderson et al. 2012), which anchor Baltic bloom populations in their respective locations (Hakanen et al. 2012, Tahvanainen et al. 2012). The life cycle of *A. peruvianum*, a heterotypic synonym of *A. ostenfeldii* (Kremp et al. 2014), was studied in detail on isolates from the Mediterranean Sea (Figueroa et al. 2008). The authors found the life cycle of *A. peruvianum* to be very complex, with various alternative options of sexual and asexual reproduction and formation of different types of cysts. This suggests that the species follows a generalist life cycle strategy, which may increase its capability to withstand variable adverse conditions and could contribute to the ongoing expansion of *A. ostenfeldii* (Van de Waal et al. 2015). Weather generalists or specialists are more successful depends on temporal and spatial environmental variability and the scale of variation. Generalists are favored by temporal variability and heterogeneous environments, whereas specialists may benefit from spatial variability and homogeneous environments (Reboud and Bell 1997, Kassen 2002, Collins 2011, Crowley et al. 2016). Strong seasonal fluctuations of temperature, light, and nutrients prevail in of the Baltic Sea, suggesting that generalist strategies are favored. Compared to the northern Baltic Sea, Mediterranean lagoons do not have strong seasonal environmental fluctuations. As such, different geographic populations could have evolved different life cycle strategies that reflect the conditions of their respective habitats, as shown for some species (Anderson 1998, Hallegraeff et al. 1998). Therefore, it is plausible that, similar to the other seasonal dinoflagellates from the northern Baltic Sea (Kremp 2000, Kremp and Anderson 2000, Kremp and Parrow 2006), *A. ostenfeldii* from the Finnish coast has a pronounced dormancy period and a narrow temperature window for germination, pointing at increased specialization. Formation of resting cysts, which are resistant to bacterial degradation during prolonged periods of cold and dark conditions, should be a prerequisite to survive the harsh winter conditions and resume vegetative growth in spring. To test this, an investigation into the regulation of life cycle transitions in a local *A. ostenfeldii* bloom population was carried out using material collected from the field and from experiments with cultured isolates. Aims of the project included defining the dormancy interval, delineating triggers for life cycle transitions and assessing how relevant genetic recombination is for the formation of resistant resting cysts (i.e., cysts that remain intact after 1 year of storage). To accomplish this, germination and encystment experiments were performed under controlled laboratory conditions. Resting cysts were isolated from sediment samples, collected repeatedly from a shallow embayment of the Åland archipelago at the SW coast of Finland. Clonal cultures were established to test encystment triggers and the relevance of genetic recombination. In addition, environmental parameters were monitored at the bloom site to assess the implications for the natural population.

## Materials and Methods

### Sediment sampling and sediment processing

To quantify cysts in the sediment and obtain a cyst slurry for all further experiments, sampling took place at the Föglö archipelago, Åland islands. Kremp et al. (2009) and Hakanen et al. (2012) described the location in detail, which corresponds to station 4 in the latter reference. The shallow sound (water depth <3 m) has a muddy bottom and is partly densely vegetated. Summer salinity is typically 6–7, the estuary is usually ice‐covered from December to April and in summer the water temperature may rise to +24°C. Replicate sediment cores were taken with a gravity corer (Limnos, Turku, Finland). The uppermost flocculent sediment layer was transferred from the middle of the cores to 50 mL centrifugation tubes. The tubes were entirely filled with sediment and stored at 4°C in the dark until further use to prevent germination of resting cysts before starting the experiments. These storage conditions were chosen to imitate winter conditions and have been applied successfully earlier (Jerney et al. 2019). Prior to the experiments ~ 5 mL sub‐samples of the sediment were diluted to 10 mL with sterile local sea water with a salinity of 6 and sonicated for 30 s on constant duty cycle with a frequency of 20 kHz (Brandelin Sonoplus sonicator HD 2200) to detach resting cysts from sediment particles. Samples were cooled with ice to avoid temperature increase during sonication and sieved afterwards to isolate the 30–76 μm fraction, containing *A. ostenfeldii* resting cysts. The material retained on the 30 μm screen was transferred into a 15 mL polypropylene centrifuge tube and diluted with filtered sterile local seawater with a salinity of 6 to obtain a cyst slurry for quantification and isolation of single cysts.

### Characterization of dormancy and seasonal germination capacity

**Figure 1:**
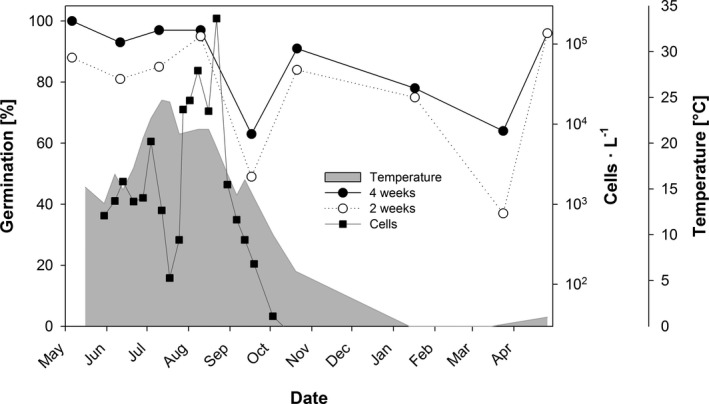
Cumulative germination of resting stages, isolated from sediment sampled repeatedly throughout the year (from May 2010 to April 2011), 2 and 4 weeks after isolation (means, *n* = 6); cell numbers of vegetative cells in the water column (logarithmic scale) and temperature at the water surface.

To characterize dormancy behavior of northern Baltic *Alexandrium ostenfeldii*, two approaches were used: (i) Germination capacity was studied with cysts isolated from freshly sampled sediment (as described above), collected throughout the annual cycle in approximately monthly intervals from May 2010 to April 2011. Germination experiments, were set up with six replicates with 7–10 cysts each. Single cysts were isolated from the cyst slurry with a glass micropipette and transferred to wells of a 12‐well tissue culture plate (Greiner Bio‐One Cellstar) filled with f/8 medium (Guillard and Ryther 1962, Guillard 1975) made from sterile local seawater with a salinity of 6. Cysts were then incubated at 16°C, 16:8 hours light:dark cycle (light intensity ~ 60 μmol photons · m^−2^ · s^−1^). After 2 and 4 weeks, the number of empty and full cysts was recorded microscopically with an inverted microscope (Leica DMI3000 B) to calculate the germination percentage. Monthly germination data were compared to *A. ostenfeldii* cell concentrations in the water to estimate the contribution of newly formed cysts to the cyst pool in the sediment and support interpretation of germination data (Fig. 1). *A. ostenfeldii* cell concentrations in the water were determined by microscopy of water samples fixed with Lugol's solution, as outlined in Hakanen et al. (2012). (ii) A second set of germination experiments was conducted using newly formed cysts sampled right after the bloom peak in September 2015. Three replicate sediment core samples were pooled, mixed, and aliquots for monthly germination experiments were stored at 4°C in the dark. The first germination experiment was set up 1 day after sampling and repeated every 2–6 weeks until April 2016 to check if cysts stored under constant conditions have the same germination capacity as freshly sampled cysts. For each experiment, one aliquot was taken from storage, processed as described above and examined for cysts. For each experiment, 50 single intact cysts were isolated into individual wells of a 96‐well plate, containing 300 μL f/8‐si medium and incubated at 16°C, 12:12 hours light:dark cycle (light intensity ~100 μmol photons · m^−2^ · s^−1^). The well plates were examined for germinated cysts 2 and 4 weeks after start of each experiment and the germination percentage was calculated as for the first approach (Figure S1 in the Supporting Information). Most viable cysts usually germinate within 2 weeks (Jerney et al. 2019), but to ascertain that all capable cysts had germinated, the well plates were re‐inspected after 4 weeks.

### Determination of temperature requirements for germination

**Figure 2:**
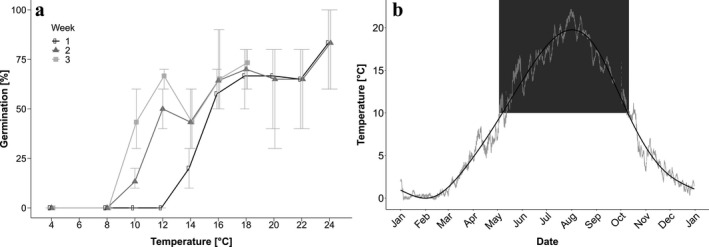
(a) Germination capacity of resting cysts collected from the field at different temperatures, after 1, 2, and 3 weeks of incubation (means ± SD,* n* = 3). (b) Sediment surface temperature at a bloom hotspot during 2017. The black line represents a generalized additive model and the gray background area indicates the temperature regulated germination interval of *Alexandrium ostenfeldii* in the Baltic Sea (restricted to temperatures above 10°C).

To determine the temperature range permitting germination of *Alexandrium ostenfeldii* cysts, a temperature gradient experiment with 10 different temperatures (4, 8, 10, 12, 14, 16, 18, 20, 22, and 24°C) was carried out in February 2018 with sediment samples collected in May 2017. Sediment was processed as described above and cysts were concentrated with the help of a density gradient using sodium polytungstate (Bolch 1997). For each temperature treatment, 30 cysts were isolated to three replicate wells (10 cysts per well) of a 96‐well tissue culture plate (Greiner Bio‐one microplates with μClear^®^ bottom and white walls), containing 300 μL of f/8‐Si. Isolation was done at 12°C room temperature to keep the cysts as cool as possible during the isolation process. Additionally, well plates were cooled on a plate heat exchanger set to 4°C, cooled by a temperature control unit (Lauda, Germany), and covered with aluminum foil during the isolation process to minimize potential light and temperature stimulation of the cysts before the start of the experiment. The cyst slurry was placed on ice and covered with aluminum foil during the isolation process for the same reason. To start the experiment, each well plate was placed on a separate plate heat exchanger connected to a temperature control unit, which was adjusted to one of the ten specific temperatures. To reduce evaporation, well plates were covered with a transparent plastic membrane, which was exchanged every second day to allow sufficient gas exchange. A custom‐made incubator with 96 white light‐emitting diodes fitted to the geometry of a 96‐well tissue culture plate was placed on top of each culture plate for illumination and adjusted to 100 μmol photons · m^−2^ · s^−1^ and a 14:10 hours light:dark cycle. The experiment lasted for 3 weeks and empty (germinated) and full (not germinated) cysts were counted with an inverted microscope (Leica DMI3000 B) once a week to calculate the percentage of germination (Fig. 2a). To relate temperature requirements for germination to seasonal temperature dynamics at the bloom site, temperature was recorded at the sediment surface (Fig. 2b) with a HOBO Pendant Temperature/Light Data Logger (Onset, Bourne, MA, USA), deployed at a water depth of 1.3 m. The data logger was attached to a steel plate, which was partly submerged in the soft sediment to prevent displacement of the logger. The temperature was recorded every 10 min from January 2017 to January 2018. Since the sampling site is very shallow and the entire water column mixed, it can be assumed that sediment surface and water surface temperatures are very similar. Therefore, temperature was measured at the water surface from 2010 to 2011.

### Encystment experiments

To define triggers and the relevance of genetic recombination for cyst formation, experiments were carried out testing the effects of different nutrient and temperature conditions on cultured clonal strains and crosses of clonal strains. Cultured clonal isolates of *A. ostenfeldii* used in these experiments were established in June to August 2015 (Jerney et al. 2019). Cultures were grown in 50 mL tissue culture flasks, filled with 40 mL f/2‐Si enriched local sea water at salinity of 6, 16°C–20°C, 100 μmol photons · m^−2^ · s^−1^ and a 14:10 hours light:dark cycle until the beginning of the experiment. The five experimental cultures were established from different sampling dates to reduce the risk of choosing sibling strains.

**Figure 3:**
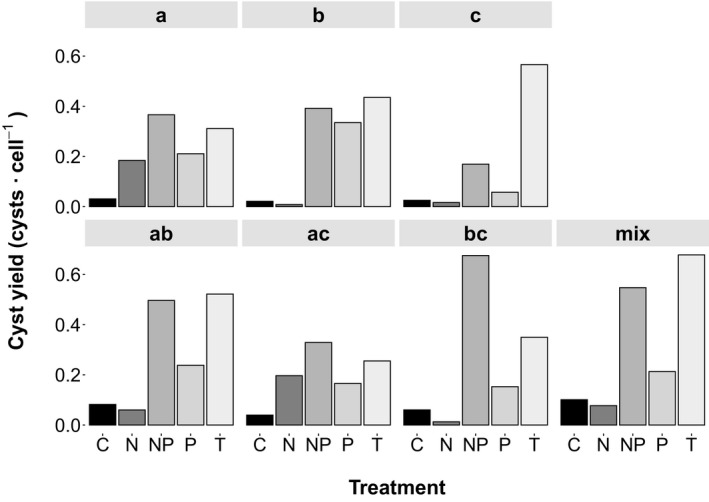
Cyst yields (number of harvested cysts at the end of the experiment divided by the maximum cell number during the growth period) of *Alexandrium ostenfeldii* clones and mixes of clones at different encystment treatments: Control (C), nitrogen limitation (N), phosphorus limitation (P), combined nitrogen and phosphorus limitation (NP), and reduced temperature (T). Single strains (a, b, and c) are shown in the upper row, combinations of two (ab, ac, and bc) and five strains (mix) in the lower row.

Single strains AOB202 (a), AOB348 (b), AOB504 (c), a combination of two strains (ab, ac, and bc), and a mix of five strains (mix = a, b, c, AOB413, and AOB329) were used for all encystment treatments to account for a potential requirement of genetic recombination. Inoculum cultures for the different experiments were grown in 500 mL of f/2‐Si medium at 16°C to reach concentrations of ~10,000 cells · mL^**−**1^ (exponential phase). Triplicate experimental units of 200 mL were inoculated with equal amounts of single strains and mixes at a starting concentration of ~1,000 cells · mL^**−**1^. Encystment was tested at 16°C with f/2‐Si medium, serving as control (C) and medium with reduced nitrate (N), phosphate (P), or both nutrients (NP) to 10% of the f/2‐Si level. In addition, one temperature treatment was tested, inoculating experimental cultures, pre‐grown in f/2‐Si medium at 16°C, into 10°C f/2‐Si medium (T). To record cell and cyst dynamics in the experimental cultures, culture flasks were gently shaken for a homogeneous cell distribution and 1.1 mL of sample was taken into an Eppendorf vial, preserved with a drop of neutral Lugol's solution. Samples were taken twice a week and cells and cysts were counted using a 1 mL Sedgewick Rafter counting chamber and an inverted microscope (Leica DMI3000 B). After 6 weeks of regular sampling, the experiments were terminated and cysts that had accumulated at the bottom of the flasks were harvested. The majority of the medium was aspirated and culture flasks and the remaining cyst slurry were shaken roughly to detach sedimented cysts from the flask bottom. Replicates were pooled into a 50 mL glass flask and sonicated for 1 min to destroy remaining cells. Afterwards cysts were concentrated with a 30 μm sieve, rinsed with filtered seawater, and transferred to 50 mL centrifugation tubes, which were filled up to 45 mL with filtered seawater. Total cysts formed in experimental flasks were enumerated from these samples and cyst yields were calculated by dividing the number of harvested cysts at the end of the experiment by the maximum cell number recorded during the growth period (Fig. 3). Another set of subsamples was transferred to 2 mL cryovials, covered with a 10 μm mesh net, closed with a drilled cap and submerged bottom‐up in anoxic sediment, which was stored at 4°C in the dark as described by Anderson et al. (2003) for later use.

**Figure 4:**
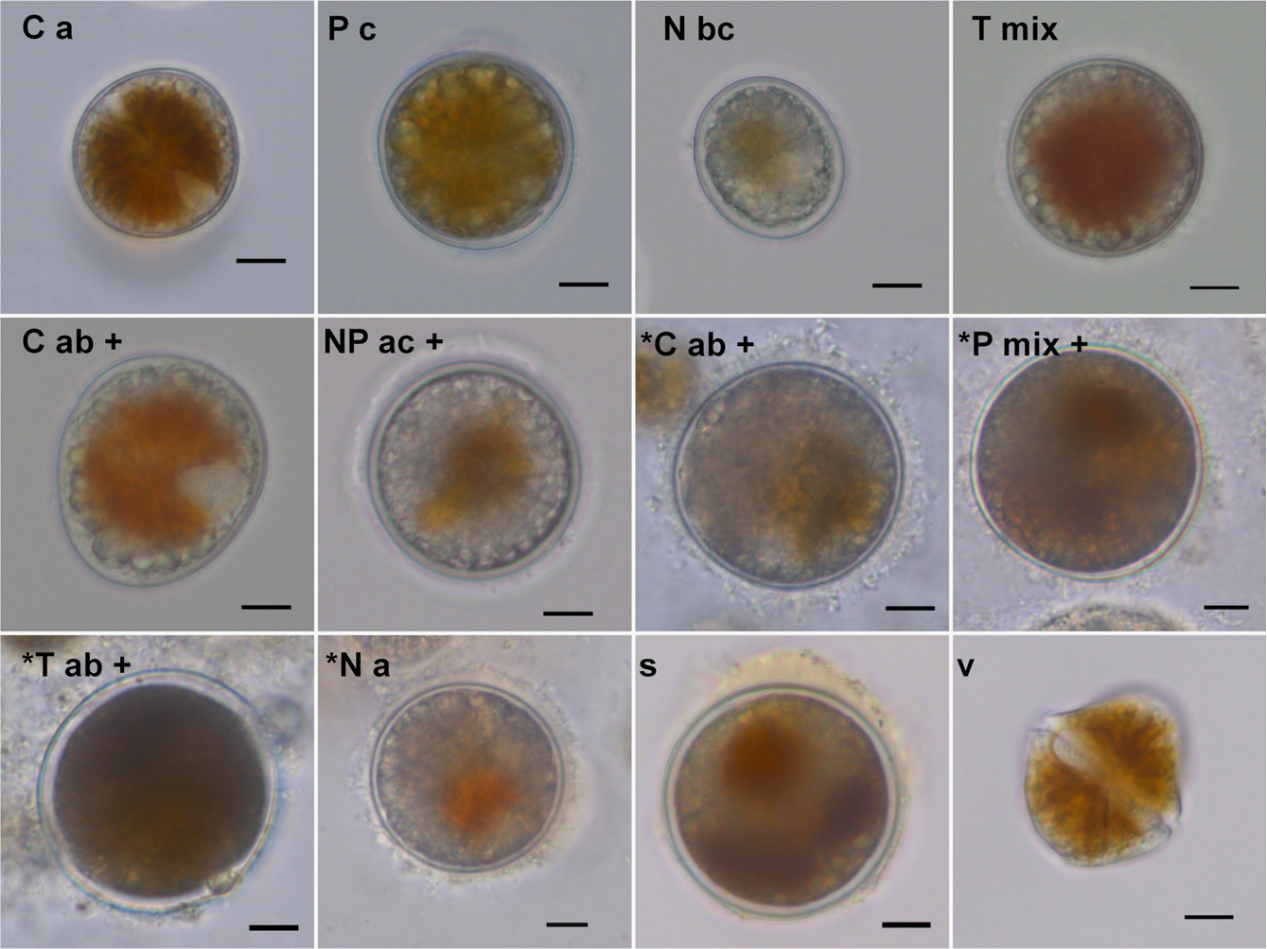
Morphological variability of *Alexandrium ostenfeldii* resting stages before and after storage for 1 year at 4°C in the dark. Capital letters indicate the treatments: Control (C), nitrogen limitation (N), phosphorus limitation (P), combined nitrogen and phosphorus limitation (NP), and reduced temperature (T). Small letters indicate single strains (a and c) or combinations of two (ab, ac, and bc) and five strains (mix). Micrographs taken after 1 year of storage at 4°C are labeled with a star (*) and successful germination with a plus symbol (+). Typical cyst found in sediment samples from Föglö archipelago (s) and a vegetative cell, grown at control conditions (v). Scale bars 10 μm. [Color figure can be viewed at https://www.wileyonlinelibrary.com]

To see whether resting cysts produced under different conditions differ morphologically from each other, micrographs were taken with a digital camera (Leica DFC490) attached to an inverted microscope (Leica DMI3000 B) after harvesting cysts at the end of the encystment experiment. Micrographs of 30 cysts from single strains and strain combinations of each treatment were inspected and compared to pictures taken after 1 year of storage (Fig. 4). Pale, disintegrated cysts with an irregular outline were considered degraded after storage, indicating that they were not resistant to bacterial degradation.

### Germination of experimentally produced cysts

**Figure 5:**
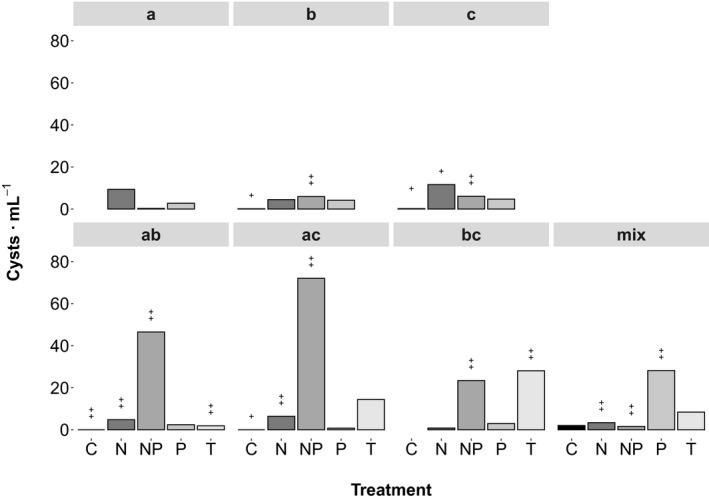
Concentration of resting cysts per ml of initial culture medium, produced in the laboratory, by different strains and strain combinations, after 1 year of storage (4°C, dark). The encystment conditions were as follows: Control (C), nitrogen limitation (N), phosphorus limitation (P), combined nitrogen and phosphorus limitation (NP), and reduced temperature (T). Single strains (a, b, and c) are shown in the upper row and combinations of two (ab, ac, and bc) and five strains (mix) in the lower row. Crosses above the bars indicate if vegetative cells were present 1 week (+) and 2 weeks (++) after re‐exposure to suitable growth conditions.

A first germination experiment was set up two days after termination of the encystment experiment, to test if internal maturation is necessary before resting cysts can germinate. For this purpose, 2 mL cryovials were taken from the anoxic storage, re‐suspended in sterile filtered sea water and 1 mL of the resulting cyst slurries were transferred to replicate 24‐well plates, containing f/8‐Si medium. Cysts were incubated for 14 d at 16°C at a light intensity of ~ 70 μmol photons m^−2^ s^−1^, 14:10 hours light:dark cycle. Germination experiments were repeated weekly or biweekly for 2 months. Cysts produced by all single strains, as well as the five strain mixes from the reduced NP and the T treatment were included in this experiment (Figure S2 in the Supporting Information). After 1 year of storage at 4°C in the dark, cyst concentrations in storage containers were determined again and germination capacity was tested as described above, though incubations were carried out in smaller volumes in eight replicate wells of a 96‐well plate, containing 200 μL f/2‐Si medium. Wells were scored positive for germination when swimming cells were observed after 1 and 2 weeks (Fig. 5).

### Statistics

Data exploration, basic calculations, and plotting of graphs were done with R (R Core Team 2018), RStudio (RStudio Team 2015), and Sigma Plot V14.0 (Systat Software, San Jose, CA, USA). Smoothed conditional means were added to the sediment surface temperature data in Rstudio with the help of the ggplot2 package (Wickham 2009) using a generalized additive model from the nlme package (Pinheiro et al. 2019) as a smoothing method.

## Results

### Seasonal germination and dormancy dynamics

*A. ostenfeldii* resting cysts germinated throughout the year from freshly sampled sediment between May 2010 and 2011 (Fig. 1). The final germination success was above 90% from May to August and in November, after 4 weeks of incubation. Shortly after the bloom peak in the end of August, germination declined to 49% but increased to 63% in September. Slightly reduced germination was also observed in February and April, but the proportion of germinated cysts remained above ca. 60%, after 4 weeks of incubation throughout the year. Dormancy dynamics was not coupled with seasonal dynamics of cells in the water column. Cells appeared at the end of May, when water temperature was above 13°C and cell numbers remained low (<5,000 cells L^−1^) until June. A maximum temperature of 24.7°C was measured in July and temperature remained around 20°C during a bloom, developing in August when cell concentrations rose to approximately 50,000 cells · L^−1^. In August, peak concentrations of >200,000 cells· L^−1^ were measured, before the cell concentration dropped suddenly below 2,000 cells· L^−1^ at the end of August during a decreasing temperature trend (around 16°C). Cells disappeared from the water completely in October, when the temperature went below 10°C.

Germination of *A. ostenfeldii* resting cysts was not inhibited endogenously during the winter months, as shown by successive biweekly to 6‐weekly incubation experiments with cysts collected shortly after the bloom peak in September 2015 (Figure S1). Germination was possible for more than 70% of the cysts, within 4 weeks of incubation, throughout the winter months until March 2016, although reduced to ca. 50% shortly after the bloom peak in September 2015.

### Temperature window for germination

Germination of *Alexandrium ostenfeldii* was completely inhibited by temperatures below 10°C (Fig. 2a). Delayed germination occurred at 10°C conditions with a mean cumulative germination below 50%. Between 10°C and 14°C germination percentage increased, but was still lower compared to 16°C and 24°C, where most viable cysts germinated within 1 week. The highest germination success of more than 75% on average was recorded at 24°C. Longer monitoring periods might have eventually revealed higher germination rates at lower temperatures but the experiments were terminated after 3 weeks because few additional cysts germinating even later were considered ecologically less relevant for bloom formation in nature.

As depicted in Figure 2b, the water temperature at the sediment surface of our sampling location showed a strong seasonal variation between 0°C in February and 22°C at the end of July. The temperature threshold of 10°C, for germination of *A. ostenfeldii,* was exceeded for the first time in the beginning of May, and temperature fell below this value in October, which allows cysts to contribute to the bloom inoculum over a period of 6 months, from May to October.

### Regulation of encystment

Encystment experiments with cultured *A. ostenfeldii* isolates showed that cyst formation is regulated by multiple factors. NP and T resulted in highest cyst yields in clonal cultures and strain combinations. The highest yield of 0.67 cysts per vegetative cell was reached in the mix, treatment T and in the bc strain combination, treatment NP (Fig. 3). T induced the highest cyst yield for single strains b and c, strain combination ab and the mix. In contrast, NP triggered highest cyst yields for strain a and the strain combinations of ac and bc. Treatment P led to low or intermediate cyst yields for all strains and strain combinations, ranging between 0.15 (bc) and 0.33 (b) cysts per vegetative cell. The least effective trigger for cyst formation was N (strains b, c, ab, bc, and mix). In two strain pairs (ab and bc) and the mix, cyst yields at NP and T were higher than in single strains.

### Morphology, survival, and germination capacity of experimentally produced cysts

Cyst morphologies varied substantially between the treatments N, P, NP, and T (Fig. 4). No pronounced morphological differences related to single strains or strain combinations were found by visual inspection and cell size measurements (data not shown). Variable cyst shapes (spherical to slightly elongated), cyst wall thicknesses, and cyst sizes were observed. Reduced pigmentation and the occurrence of a red accumulation body were associated with cyst formation triggers: Cysts formed under C and T were strongly pigmented and had on average few thick‐walled cysts (14% and 12.5%). More thick‐walled cysts were formed under N, NP, and P (23, 27 and 23.5%), but in total the majority of cysts were thin‐walled (80%). Cysts formed at N and P had thicker walls compared to the other treatments, despite centered, weak pigmentation. To see whether the cyst morphology changes over time, micrographs of cysts before and after (Fig. 4, star symbol) storage were compared. Cyst morphology was different from newly formed cysts after 1 year of storage because mainly spherical and no elongated cysts were found. Although there were many degrading, pale cysts, most of the intact cysts had a strong pigmentation.

To compare the germination capacity of freshly produced and stored cysts, germination experiments were set up 2 days and 1 year after harvesting cysts of the encystment experiment. Around 40%–70% of the freshly produced cysts germinated within 7 d, independent of the encystment trigger and strain combination (Figure S1). Cysts that resembled wild cysts (Fig. 4s) had the best germination capacity after storage, independent of the treatment. Compared to single strains strain combinations produced higher numbers of resistant cysts, with a higher germination capacity after storage (Fig. 5). NP and combinations of two strains resulted in the highest number of cysts after storage: 1.14% (ac) and 0.87% (ab) of cells formed cysts, equaling 72 cysts · mL^−1^ (ac) and 47 cysts · mL^−1^ (ab). Fewer cysts (20–30 cysts · mL^−1^) were formed by P (mix), NP, and T (bc), equaling 0.55, 0.43, and 3.1% of cysts per cell. A high percentage (3.1%) of cysts found in the bc strain mix of treatment T is related to low cell numbers due to slow growth at the low temperature. The number of viable cysts was much lower for single strains after 1 year of storage (below 12 cysts · mL^−1^, or 0.2 % cysts per cell) and only cysts produced by single strains b and c germinated and grew thereafter (indicated by two crosses above bars in Fig. 5).

## Discussion

This study demonstrates that the majority of the Baltic *A. ostenfeldii* resting stages are quiescent throughout the year. Newly formed resting cysts have a very short maturation period before possible germination and no pronounced dormancy interval, as it is typical in seasonal bloom forming Baltic dinoflagellates (Kremp 2000, Kremp and Anderson 2000, Kremp and Parrow 2006). Inhibition of quiescent cysts occurred as a result of temperatures below 10°C in our experiments. Suitable germination temperatures, corresponding to the occurrence of the species in the water column, lasted from May to October. Temperature reduction to 10°C and NP limitation of nitrogen and phosphorus were the strongest encystment triggers. More resistant cysts were formed when multiple strains were combined, especially when cyst formation was triggered by combined phosphorus and nitrogen limitation. In addition, a variety of treatment‐related cyst morphologies were observed.

### Regulation of dormancy and germination

Tightly linked dormancy and temperature control of cyst germination often determine the growth phase of dinoflagellates in seasonal habitats (Ellegaard et al. 1998, Kim and Han 2000, Anderson and Rengefors 2006). The strong seasonality of the Baltic Sea prevents growth of warm‐water‐adapted *A. ostenfeldii* during the winter months and blooms usually occur between July and August (Hakanen et al. 2012). Accordingly, the expectations for this study included finding a winter dormancy period and a summer germination temperature interval. However, results of this study reveal a different germination strategy for the species: a lack of dormancy during the winter months, coupled with moderate temperature control, preventing germination at water temperatures below 10°C. This temperature control can be considered moderate, compared to other seasonal dinoflagellates, which have more narrow temperature ranges controlling germination (Rengefors and Anderson 1998, Kremp and Anderson 2000). Similarly, a wide range of temperatures (12°C–28°C) allowed high germination rates of *A. catenella (= A. tamarense)* from Thau lagoon, France (Genovesi et al. 2009). The authors suggested that the observed seasonal germination pattern was independent of temperature fluctuations. The study presented here shows that the majority of *A. ostenfeldii* cysts in the sediment are quiescent and ready to germinate throughout the year, as soon as favorable conditions are provided. Conditions are considered favorable if they support vegetative growth and reproduction (e.g., temperatures above 10°C, sufficient light and nutrients). Reduced germination capacity of the cyst population was detected shortly after the bloom peaked in September 2011 (Fig. 1) and 2015 (Figure S1), which could reflect the recent deposition of newly formed, immature cysts decreasing the fraction of total cysts capable of germination in the investigated pool. The fact that the same pattern was found in both years supports this interpretation. The slightly reduced germination capacity in April 2011 is inconsistent with the rest of the data due to high germination success earlier in winter and in May 2012. This inconsistency possibly reflects the natural variability of germination in the population, which can be high, spatially and temporally, when not all genotypes follow the same strategy (Anderson 1998). Alternatively, several germination strategies might exist in the Baltic *A. ostenfeldii* population and partly explain the observed seasonal variation. Continuous germination after a short maturation period and germination controlled by an circannual internal rhythm (preventing germination of dormant cysts) could both exist as alternative strategies in the same population, as suggested for other seasonal *Alexandrium* species (Perez et al. 2002). Coexistence of several germination (emergence) strategies was proposed to result from different levels of temporal environmental variability (De Stasio and Hairston 1992, Martínez‐García and Tarnita 2017) and can ensure survival in case the majority of a population germinates, but is lost afterwards due to unfavorable environmental conditions (Anderson 1998).

As reported for *A. catenella (= A. tamarense)* from Thau lagoon, France, (Genovesi et al. 2009), *A. ostenfeldii* in this study required a very short maturation period after cysts were formed, before they could germinate again. The interpretation of field data is supported by the results of laboratory experiments which showed that cysts derived from encystment experiment (Fig. 3) were able to germinate at a high rate within 7–14 d once suitable conditions were restored (Figure S1). Interestingly, very short maturation periods of 10–20 d were reported for a Chinese *A. ostenfeldii* strain from Bohai Sea (Gu 2011) which is genetically closely related to the Baltic Sea population (Kremp et al. 2014), but experiences different habitat conditions. In addition, a short maturation period of less than 10 d for Mediterranean *A. ostenfeldii* sensu Kremp et al. (2014) strains was reported (Figueroa et al. 2008), suggesting that the duration of maturation in *A. ostenfeldii* may, to some extent, be genetically predetermined.

In contrast to Baltic *A. ostenfeldii*, prolonged dormancy periods, lasting several months, can inhibit germination in other seasonal *Alexandrium* spp. (e.g., Anderson 1980, Kim et al. 2002, Mardones et al. 2016, Fischer et al. 2018). Also resting cysts of Mediterranean populations of *A. ostenfeldii* sensu Kremp et al. (2014) have a dormancy period of numerous months (Figueroa et al. 2008) although habitat conditions at the Catalan coast are stable compared to the Baltic Sea. Correspondingly, variable dormancy periods of one and the same species, but different geographic isolates, were found for *A. catenella* (Hallegraeff et al. 1998). According to Hallegraeff et al. (1998), different ecological roles of cysts, such as overwintering strategy versus rapid cycling between benthos and plankton, might be responsible for varying cyst dormancy requirements.

Temperature is an important factor in controlling dinoflagellate cyst germination by maintaining quiescence for extended periods, determining the duration of dormancy after cyst formation, synchronizing germination or initiating the excystment process (Anderson 1998 and references therein). The data presented here show that a defined temperature window for germination of Baltic *Alexandrium ostenfeldii* exists*,* ranging from 10°C to 24°C (Fig. 2a). At lower temperatures (10°C–14°C), the average germination success is reduced, which indicates that temperature functions as environmental filter, preventing germination of some part of the cyst population. A broad temperature range permitting germination is in line with earlier findings, reporting that temperature does not affect germination success between 16°C and 20°C (Jerney et al. 2019). Lower temperatures reduced germination of *A. tamarense* from Irish coastal waters, whereas excystment of *A. minutum* from the same habitat showed no temperature effect with almost equal success between 5°C and 25°C (Ní Rathaille and Raine 2011).

The temperature requirements of Baltic *A. ostenfeldii* restrict germination to the period between May and October (Fig. 2b), which is when cells of *A. ostenfeldii* can indeed be found in the water column (Fig. 1; Hakanen et al. 2012). A comparable temperature range was determined for the Chinese isolate from Bohai Sea (Gu 2011) and also the quicker germination at higher temperatures described by these authors is similar to our results (Fig 2a). These similarities further emphasize the close relationship of Baltic and Bohai Sea populations indicated by genetic data (Sildever et al. 2019).

### Triggers of cyst formation

T and NP are the most effective triggers of northern Baltic *A. ostenfeldii* encystment (Fig. 3). Significant cooling of surface water—that is rapid decrease of temperature by several degrees—should be perceived by the organism as a signal of impending unfavorable growth conditions and trigger a protective response. In the northern Baltic, increasing temperature induced formation of dormant resting cysts of several cold water dinoflagellates (Kremp et al. 2009) which usually bloom in spring (Kremp and Heiskanen 1999). Thus, it is anticipated that a respective reverse temperature change triggers encystment of a species, which forms blooms in summer (Hakanen et al. 2012). Limiting concentrations of inorganic N and P do not seem to be directly related to bloom termination involving cyst formation in the field (Hakanen et al. 2012), but might represent an alternative strategy to ensure the deposition of resistant resting cysts in case temperature would not drop early enough before other conditions become unfavorable. Single nutrient limitation was less effective in triggering cyst formation than the other treatments, and P resulted in higher cyst yields than N. Despite the many similarities of the Chinese isolate with Baltic populations, the effect of P is inconsistent with the findings of Gu (2011), showing a higher cyst induction of the Chinese strain by N compared to P.

All single strains and strain combinations formed resting cysts (Fig. 3), which indicates that resting cysts can be formed with and without genetic recombination (i.e., by heterothallic and homothallic sexual reproduction). In contrast, planozygote and cyst formation of *Alexandrium minutum* was, for example, reported to be affected by low P/N ratios, low concentration of both nutrients and by salinity and temperature (Figueroa et al. 2011). Cyst yields of this study were higher in strain mixes than in single strains, possibly indicating heterothallic sexual reproduction, but the effect was not dramatic. Conclusions about the rate of genetic recombination of Baltic *A. ostenfeldii* strains remain speculative at this point because the development of single cells was not followed to trace and quantify zygote formation. Variable mating systems, with homothallic and heterothallic sexuality occurring in one and the same species, have been described for other dinoflagellates (Figueroa et al. 2010) and also the formation of dormant resting cysts without sexual reproduction might be more common than previously assumed (Kremp and Parrow 2006). Zygote formation by clonal strains was observed in *A. ostenfeldii* cultures previously (Jensen and Moestrup 1997, Figueroa et al. 2008, Gu 2011) and underlines the assumption that many cases of sexuality reported in the literature may be the result of self‐fertilization (Jensen and Moestrup 1997).

Observations from this study showed that Baltic *A. ostenfeldii* form a large diversity of cyst types, which is in line with earlier findings (Figueroa et al. 2008). The morphological characteristics of cysts, such as shape, thickness of the cyst wall, pigmentation, and size, seemed to be associated with encystment triggers rather than sexuality or the strain identity. In addition, the morphology of freshly produced cysts did not lead to conclusions about their resistance, since cysts of variable morphology showed different storage and germination capacities. Though thick‐walled cysts are typically regarded as resistant resting cysts, relatively thin‐walled cysts germinated in experiments after 1 year of storage (Fig. 4). Thin‐walled cysts of other dinoflagellates have been demonstrated to function as dormant survival cyst, preserving the cell from degradation (Kremp 2000).

Results of the 1‐year storage experiment, showing higher resistance and germination capacity of combined strain incubations (Fig. 5), suggest that genetic recombination could increase survival and resistance of cysts. Single strains tend to produce more temporary cysts, as documented in other studies (Jensen and Moestrup 1997, Figueroa et al. 2008). This study therefore supports the view that sexual reproduction and cyst formation can be independent processes (Bravo et al. 2014). The decoupling of cyst formation and sexual reproduction could facilitate increased asexual reproduction, which was proposed to become selected in marginal environments (Eckert 2002). When analyzing the genetic structure of several Baltic *Alexandrium ostenfeldii* populations, a previous study reported significant multilocus disequilibrium, indicating reduced sexual reproduction (Tahvanainen et al. 2012).

### The role of temporary encystment

A high germination capacity throughout the year is unexpected for Baltic *A. ostenfeldii* as it seems to be a risky strategy in a temporally variable environment with frequent short‐term fluctuations, in addition to seasonal variation. A large part of the population could be lost if favorable temperature conditions do not persist long enough for survival and reproduction. As shown in Figure 2b, temperature fluctuations of several degrees happen frequently throughout the year and it is presumable that temperature can rise above 10°C already in spring, but decrease thereafter again below this value. Repeated losses of a large fraction of cells germinating at the wrong time could exploit the seedbank and have severe long‐term consequences, eventually resulting in extinction of a population. Prevention of germination under favorable conditions could reduce this risk, in the event that environmental conditions turn unfavorable again (Donohue et al. 2010). Large temporal variance in survival and reproductive stress favors dormancy, which is commonly thought of as an adaptation to variable environments (Venable and Brown 1988). On the other hand, dormancy would be less important if only a small fraction of cysts, located in the uppermost oxygenated sediment layer of the seed bank, can germinate (Anderson et al. 2012) or if environmental conditions do not fluctuate frequently. Cyst flux data are not available from the Föglö area, but the uppermost centimeter of the sediment is usually well oxygenated and flocculent, indicating that a large fraction of cysts could germinate under suitable conditions. Alternatively, the germination of other seasonal *Alexandrium* populations, shown to be independent of temperature and an endogenous clock, was suggested to be governed by secondary dormancy (Ní Rathaille and Raine 2011). Secondary dormancy is mediated environmentally and can prevent germination of mature cysts during periods that are favorable for germination but are not favorable for growth (Ní Rathaille and Raine 2011), whereas an endogenous annual clock regulates germination internally, irrespective of environmental conditions (Anderson and Keafer 1987). However, if life history transitions (encystment and germination) are initiated rapidly and are coupled with a quick turnover between the benthic and the pelagic phase via temporary encystment, survival, and reproductive success might not be compromised in variable environments; thus, dormancy becomes less relevant. Temporary asexual cysts can be frequently observed in ageing cultures of Baltic *A. ostenfeldii* and were also reported in Mediterranean and Danish strains (Jensen and Moestrup 1997, Figueroa et al. 2008).

### Generalist life cycle strategy and global warming

With respect to their food or habitat preferences, species are considered generalists if they have broad preferences and specialists are defined as organisms with restricted use of habitats or resources (Ricklefs 1999). To expand this terminology to the life cycle, a generalist can be defined as species with broad range of possible life cycle transitions and triggers inducing them, whereas a specialist is restricted to a specific strategy and a specific trigger. According to this definition, for example, *Pseudo‐nitzschia* spp. can be regarded as specialists because members of this genus follow one very specific reproduction strategy without known alternative life cycle options (von Dassow and Montresor 2011). In contrast, the versatility of encystment triggers, cyst types as well as germination and survival capacities of produced cysts support the view that Baltic *A. ostenfeldii* follows a generalist life cycle strategy. Although cyst formation is required as an overwintering strategy, pointing at increased specialization, alternative life cycle routes exist and transitions are regulated by a broad range of triggers. In addition to flexible germination behavior, Baltic *A. ostenfeldii* populations are characterized by high plasticity and a great adaptation potential, based on large genotypic and phenotypic diversity, which most likely aids its adaptation and persistence under predicted future climate conditions (Kremp et al. 2016, Jerney et al. 2019). Due to global warming, an overall reduction of sea surface salinity and increase of mean sea surface temperature are expected in the future (e.g., Meier et al. 2012), which could aid expansion of this species (Kremp et al. 2012). Alternatively, expansion of *A. ostenfeldii* could be limited by increasing hypoxia in coastal zones of the Baltic Sea (Conley et al. 2011), since germination of dinoflagellates requires oxygen (e.g., Rengefors and Anderson 1998, Kremp and Anderson 2000). Recent studies showed that the beneficial effect of higher temperature on growth might be compromised by decreased salinity (Jerney et al. 2019), which makes predictions about future bloom development challenging. Furthermore, bloom formation depends on many other variables, like competition with other co‐occurring phytoplankton, predation pressure or the availability of nutrients and convincing evidence that climate change will enhance the growth of harmful algal bloom species, like *Alexandrium*, over the far larger pool of competing other phytoplankton species is lacking (Wells et al. 2015).

## Conclusions

This study concludes that the majority of the *A. ostenfeldii* resting cysts are quiescent throughout the year and germination is not inhibited by endogenous regulation during winter. Results indicate that newly formed cysts undergo a short maturation period of around 1 month, after which they are ready to germinate as soon as conditions become favorable. Temperature regulated germination is possible above 10°C and accelerated above 16°C. In shallow inlets of the Föglö archipelago inhabited by *A. ostenfeldii*, temperature of the sediment surface shows a strong seasonal variation which restricts germination and the occurrence of vegetative cells in the water column up to 6 months (from May to October). In the future, this germination period will likely expand due to an increase in the mean sea surface temperature by global warming (Meier et al. 2012). At the end of the growth season, cyst formation is likely triggered by a combined effect of nutrient limitation and a temperature drop below 10°C. This study shows that genetic recombination is not strictly required for the formation of resting cysts, but it strongly enhances formation of resistant cysts and resurrection capacity after a resting period. Life cycle transitions of *A. ostenfeldii* seem to be very flexible, indicating that this species follows a generalist life cycle strategy. A generalist strategy might support this species persistence and possibly expansion in geographically and ecologically marginal environments in the future if higher temperatures facilitate a longer growth season. Populations in marginal ecosystems are often isolated and under extreme selection pressures, like exposure to harsh physical conditions, environmental gradients, and anthropogenic impacts, resulting in anomalous genetics (Johannesson and André 2006). To fully understand the bloom dynamics of this species, quantification of cyst fluxes (including sedimentation and re‐suspension rates) should be assessed in the future. Understanding the life cycle of resting cyst forming phytoplankton species and mechanisms affecting life cycle transitions is crucial because it will affect the interpretation of cyst records from the past. As demonstrated recently, past cyst records do not always match phytoplankton monitoring data (Kremp et al. 2018), which could be related to different modes of cyst formation, affecting their resistance to bacterial degradation. Another important aspect of alternative life cycle strategies is the sedimentation of different types of cysts and their fate—either burial of resistant resting cysts or degradation of cells and temporary cysts—which will affect biogeochemistry of the sediment and nutrient fluxes (Spilling and Lindström 2008). Once the life cycle of species is fully understood, modeling studies can be carried out to follow bloom trends and predict future bloom development (Lee et al. 2018).

## Conflict of Interest

The authors declare no conflicts of interest.

## Author Contributions

Conceived and designed the experiments: JJ, AK, and SS. Carried out sampling: JJ, PH. Performed the experiments: JJ, PH, SAA, AK, SS. Analyzed the data: JJ. JJ led the writing of the manuscript with input from all authors.

## Data Sharing and Data Accessibility

Data will be accessible at Dryad.

## Protection of Human Subjects and Animals in Research

Not relevant for this study.

## Supporting information


**Figure S1.** Germination of *Alexandrium ostenfeldii* cysts, isolated from sediment sampled in September 2015 during a bloom peak (~11 × 10^3^ cells · L^**−**1^) and stored afterwards in aliquots, at 4°C in the dark. Every 2–6 weeks 50 cysts were isolated and inoculated at suitable growth conditions to check for germination two and four weeks later.

**Figure S2.** Germination percentage of resting stages, produced under controlled conditions in the laboratory, plotted against the number of storage days. Germination experiments were started two days after harvesting of cysts and repeated every 7–14 d (means, *n* = 3). Cysts were stored at 4°C in the dark and germination success was recorded after seven days of incubation. Cyst formation was triggered by the following treatments: Nitrogen and phosphorus limitation for single strains (a, b, c) and the combination of five strains (mix) and a drop of temperature for the combination of five strains (T mix).
