# Supplementary figures and images for: Generalist Life Cycle Aids Persistence of Alexandrium ostenfeldii (Dinophyceae) in Seasonal Coastal Habitats of the Baltic Sea
Source: J Phycol. 2019 Oct 6;55(6):1226–38. doi: 10.1111/jpy.12919 (PMC6916352; doi:10.1111/jpy.12919)

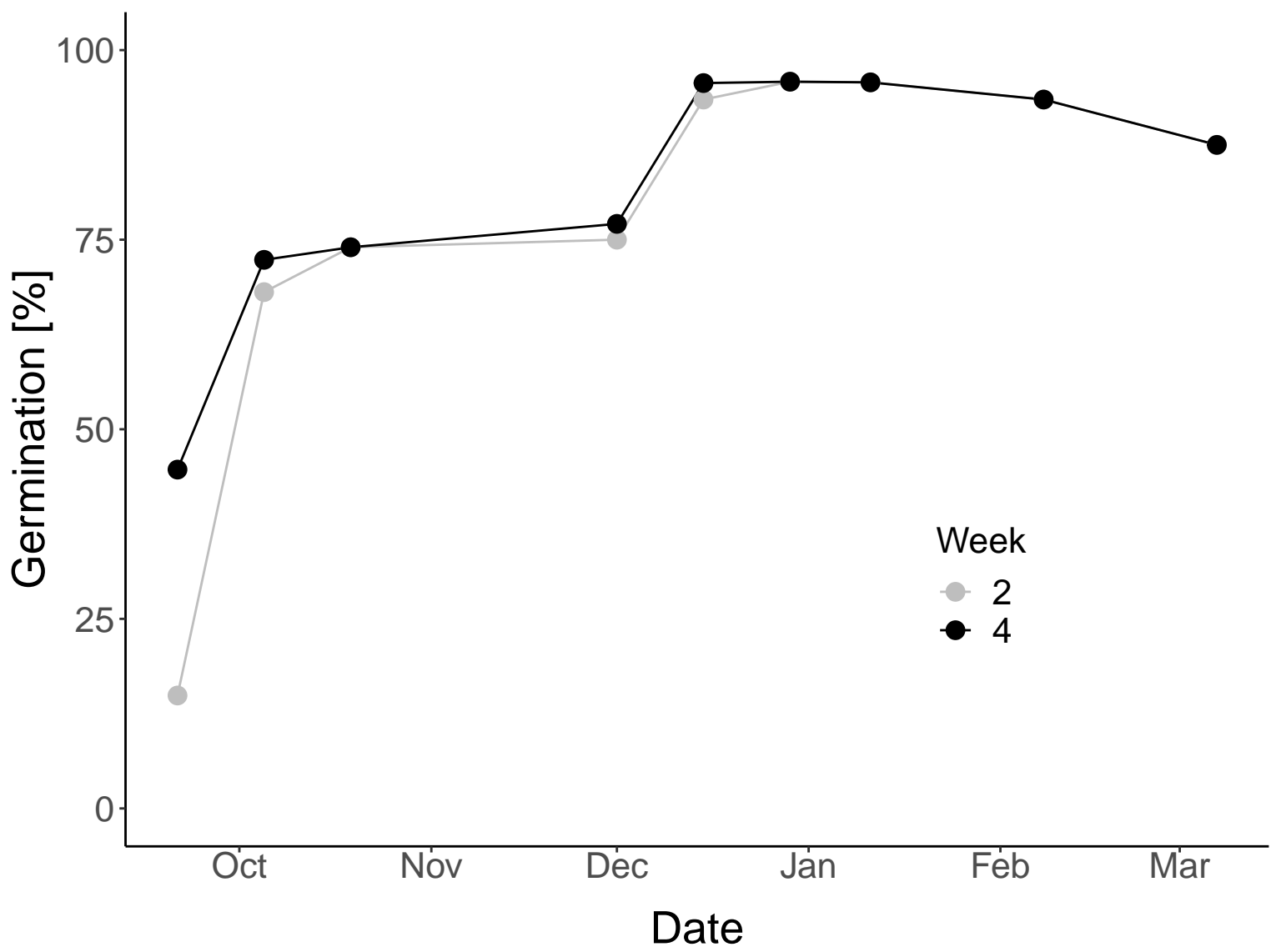

Supplement: Supplementary file 1 — Figure S1. Germination of Alexandrium ostenfeldii cysts, isolated from sediment sampled in September 2015 during a bloom peak (~11 × 103 cells · L−1) and stored afterwards in aliquots, at 4°C in the dark. Every 2–6 weeks 50 cysts were isolated and inoculated at suitable growth conditions to check for germination two and four weeks later. [file JPY-55-1226-s001.pdf]

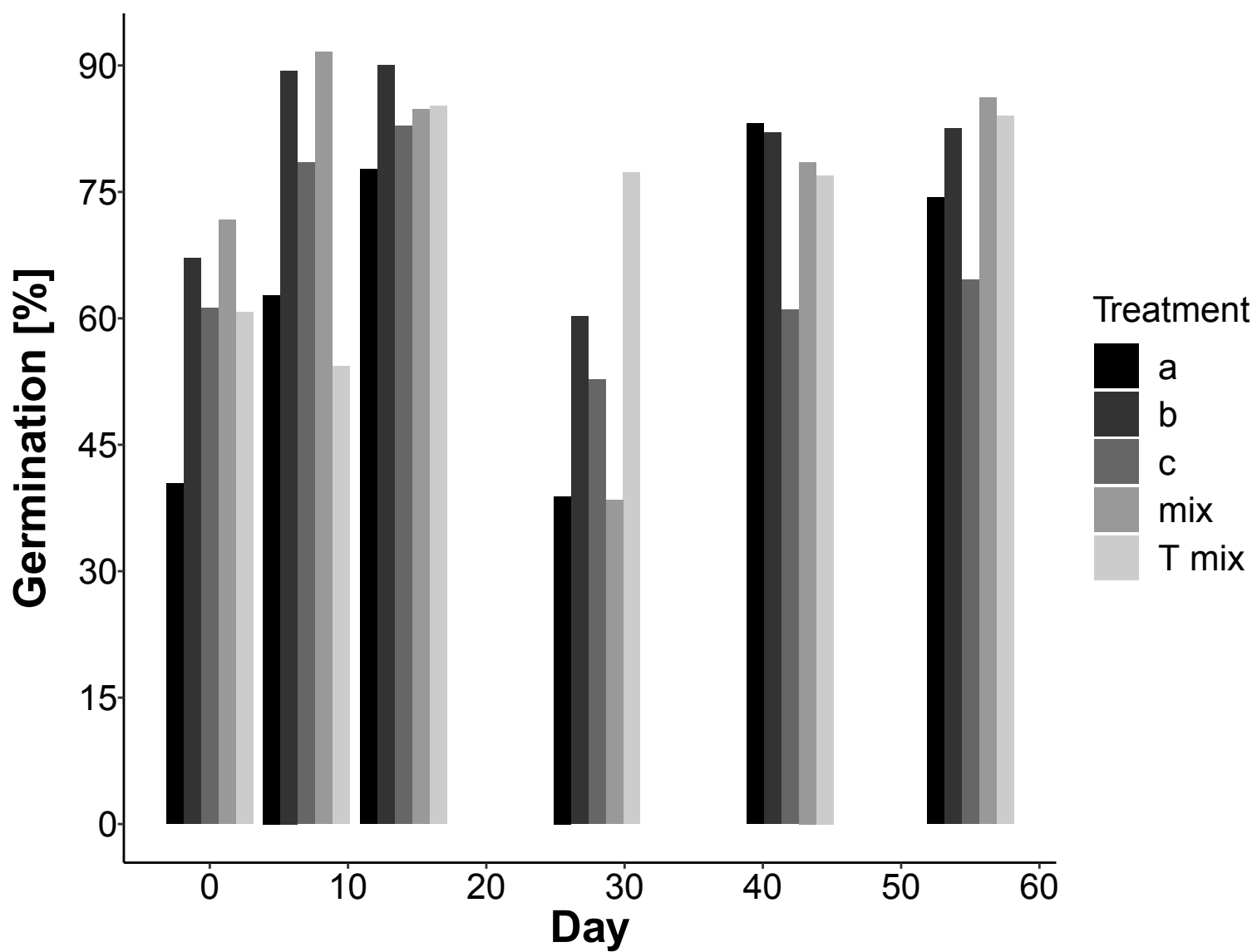

Supplement: Supplementary file 2 — Figure S2. Germination percentage of resting stages, produced under controlled conditions in the laboratory, plotted against the number of storage days. Germination experiments were started two days after harvesting of cysts and repeated every 7–14 d (means, n = 3). Cysts were stored at 4°C in the dark and germination success was recorded after seven days of incubation. Cyst formation was triggered by the following treatments: Nitrogen and phosphorus limitation for single strains (a, b, c) and the combination of five strains (mix) and a drop of temperature for the combination of five strains (T mix). [file JPY-55-1226-s002.pdf]
